# Inequities in utilization of prenatal care: a population-based study in the Canadian province of Manitoba

**DOI:** 10.1186/s12884-018-2061-1

**Published:** 2018-11-01

**Authors:** Maureen I. Heaman, Patricia J. Martens, Marni D. Brownell, Mariette J. Chartier, Kellie R. Thiessen, Shelley A. Derksen, Michael E. Helewa

**Affiliations:** 1 College of Nursing, Rady Faculty of Health Sciences, University of Manitoba, 89 Curry Place, Winnipeg, MB R3T 2N2 Canada; 2 Department of Community Health Sciences, Max Rady College of Medicine, Rady Faculty of Health Sciences, University of Manitoba, S113 - 750 Bannatyne Avenue, Winnipeg, MB R3E 0W3 Canada; 3 Manitoba Centre for Health Policy, University of Manitoba, 408-727 McDermot Avenue, Winnipeg, MB R3E 3P5 Canada; 4 Department of Obstetrics, Gynecology and Reproductive Sciences, Max Rady College of Medicine, Rady Faculty of Health Sciences, University of Manitoba, WR120-735 Notre Dame Avenue, Winnipeg, MB R3E 0L8 Canada

**Keywords:** Prenatal care, Pregnancy, Delivery of health care, Socioeconomic factors, Cohort studies

## Abstract

**Background:**

Ensuring high quality and equitable maternity services is important to promote positive pregnancy outcomes. Despite a universal health care system, previous research shows neighborhood-level inequities in utilization of prenatal care in Manitoba, Canada. The purpose of this population-based retrospective cohort study was to describe prenatal care utilization among women giving birth in Manitoba, and to determine individual-level factors associated with inadequate prenatal care.

**Methods:**

We studied women giving birth in Manitoba from 2004/05–2008/09 using data from a repository of de-identified administrative databases at the Manitoba Centre for Health Policy. The proportion of women receiving inadequate prenatal care was calculated using a utilization index. Multivariable logistic regressions were used to identify factors associated with inadequate prenatal care for the population, and for a subset with more detailed risk information.

**Results:**

Overall, 11.5% of women in Manitoba received inadequate, 51.0% intermediate, 33.3% adequate, and 4.1% intensive prenatal care (*N* = 68,132). Factors associated with inadequate prenatal care in the population-based model (*N* = 64,166) included northern or rural residence, young maternal age (at current and first birth), lone parent, parity 4 or more, short inter-pregnancy interval, receiving income assistance, and living in a low-income neighborhood. Medical conditions such as multiple birth, hypertensive disorders, antepartum hemorrhage, diabetes, and prenatal psychological distress were associated with lower odds of inadequate prenatal care. In the subset model (*N* = 55,048), the previous factors remained significant, with additional factors being maternal education less than high school, social isolation, and prenatal smoking, alcohol, and/or illicit drug use.

**Conclusion:**

The rate of inadequate prenatal care in Manitoba ranged from 10.5–12.5%, and increased significantly over the study period. Factors associated with inadequate prenatal care included geographic, demographic, socioeconomic, and pregnancy-related factors. Rates of inadequate prenatal care varied across geographic regions, indicating persistent inequities in use of prenatal care. Inadequate prenatal care was associated with several individual indicators of social disadvantage, such as low income, education less than high school, and social isolation. These findings can inform policy makers and program planners about regions and populations most at-risk for inadequate prenatal care and assist with development of initiatives to reduce inequities in utilization of prenatal care.

## Background

Prenatal care is important to achieving a healthy pregnancy and birth and positively influencing the health of the fetus and child [1]. The Marmot Review [2], “Fair Society, Healthy Lives,” emphasized the importance of ensuring high quality maternity services across the social gradient. Despite the emphasis placed on the value of prenatal care, a portion of the childbearing population continues to receive inadequate prenatal care, defined as receiving no prenatal care, initiating care later than the first trimester, or, given a first trimester start of care, receiving less than the recommended number of visits [3]. In the United States (U.S), 11.2% of women received inadequate prenatal care in 2004 [4], while in 2016, 77.1% of women began prenatal care in the first trimester of pregnancy, 4.6% began care late (in the third trimester), and 1.6% had no prenatal care, with significant disparities by race/ethnicity [5]. In Canada, national population-level data are not collected on utilization of prenatal care and therefore the rate of inadequate prenatal care is not included as an indicator in the Perinatal Health Reports published by the Public Health Agency of Canada [6, 7]. One older study reported an 8.9% rate of inadequate prenatal care in the Canadian province of Manitoba in 1987/88 [8], while another reported a rate of 6.9% from 1991 to 2000 [9], using different measures of prenatal care utilization. Given that Canada has a universal health care system, and women are not required to pay for prenatal care, these findings suggest inequities in utilization of prenatal care and the existence of barriers other than cost of care. Marmot defines inequity as an inequality or difference that is not fair or just, and is preventable and avoidable [10].

Inadequate prenatal care is a well-recognized risk factor for adverse pregnancy outcomes [11, 12]. In a study of over 28 million births in the U.S., inadequate prenatal care was associated with an increased risk of preterm birth, stillbirth, and early and late neonatal death [11]. In addition, there is growing evidence of an association between prenatal care utilization and subsequent use of postpartum care [13] and well child visits [14, 15]. Thus, efforts to reduce inequities in utilization of prenatal care may contribute to improved maternal and child outcomes. Although several studies on factors associated with inadequate prenatal care have been conducted in the U.S. and other high-income countries [16], the results are not necessarily generalizable to the Canadian population, with its different health care system and racial/ethnic composition. Only a few studies have explored use of prenatal care in the Canadian context [8, 17–22].

In previous work, members of our research team conducted a population-based ecologic study of women having singleton live births in Manitoba from 1991 to 2000 to identify neighborhood-level determinants of prenatal care utilization [9]. We found wide regional variations in the proportion of women receiving inadequate prenatal care, with rates ranging from 1.1 to 21.5% across 498 geographic areas. There was a geographic concentration of high rates of inadequate prenatal care in the inner-city of Winnipeg and in northern Manitoba, areas known to be more socio-economically deprived. After adjusting for individual characteristics of age and parity, women living in areas with the highest proportion of the population who were unemployed, Aboriginal, recent immigrants, single parent families, or having less than 9 years of education, or who lived in areas with the lowest average household income, had the highest rates of inadequate prenatal care [9]. This earlier study provided initial evidence of inequities in use of prenatal care. The purpose of the current population-based study was to expand our understanding of individual-level factors associated with inadequate prenatal care in Manitoba.

Since 2000, new initiatives with the potential to improve use of prenatal care have been implemented in Manitoba, such as the Healthy Baby program [23–25] and regulation of the profession of midwifery [26], creating a need for an updated study of prenatal care utilization. There have also been a number of improvements and additions to the databases housed in the Population Research Data Repository at the Manitoba Centre for Health Policy (MCHP) that allow researchers to significantly improve upon the approach used in the earlier population-based studies of prenatal care [8, 9]. Because physicians used to bill for provision of prenatal care using a global tariff instead of claiming reimbursement for each visit, earlier studies had to rely on hospital abstracts to identify prenatal care visits, which were abstracted from the prenatal record; these data had a high percent of missing information (12–15%), and coding of visits was restricted to one digit, therefore limiting the recorded number of prenatal care visits to a maximum of 8 (with a code of 9 indicating missing data). As of 2001, the medical claims system was revised to have physicians submit claims for reimbursement for the initial prenatal visit and each subsequent visit. Around the same time, space for coding of prenatal care visits in the discharge abstracts was increased to two digits. These changes made determination of the timing and number of prenatal care visits more accurate. In addition, earlier research in Manitoba was limited to only a few individual level variables available in the data files, such as age and parity, necessitating greater reliance on area-level variables derived from the Canadian Census. With the incorporation of data files from Healthy Child Manitoba and Manitoba Families at MCHP, individual level variables such as achievement of high school education, social risk factors (social isolation, single parent status) and health behaviors (smoking, alcohol and drug use) from the Families First screen [27] and receipt of income assistance could be studied.

The current population-based study therefore updates and extends our previous work. The objectives of this study were:

1. To describe rates of inadequate, intermediate, adequate, and intensive prenatal care utilization among women giving birth in the province of Manitoba from 2004/05 to 2008/09 and to examine trends over time;
2. To describe variation in rates of inadequate prenatal care by geographical region; and
3. To determine factors associated with inadequate prenatal care.

## Methods

### Study design, setting, and inclusion criteria

This was a population-based retrospective cohort study of all women giving birth in hospital in Manitoba over a five-year time period, from 2004/05 to 2008/09. We included women with live births, stillbirths, and singleton or multiple births, in order to provide a population-level examination of prenatal care utilization across the spectrum of types of births. In 2006, Manitoba had a population of 1,148,401 people, and the metro area population for the capital city of Winnipeg was 694,668 people [28]. There were approximately 14,000 to 15,000 births per year in Manitoba during the time frame of this study, and women received prenatal care from obstetricians (41%), family physicians (35%), midwives (4.7%) or a mix of providers (19.1%) in 2008/09 [29]. The provincial Ministry of Health provides comprehensive universal health care coverage for essentially all residents of Manitoba.

### Data sources

We analyzed data from existing administrative databases available in the Manitoba Population Research Data Repository (hereafter referred to as the Repository) housed at the MCHP in the University of Manitoba. This Repository is an extensive, person-level, linkable but de-identified collection of administrative databases for all permanent residents of Manitoba, covering both health and social services records. The validity and utility of information in the repository has been well documented [30–32]. The specific data files analyzed for this project were as follows:

- Hospital Abstracts file includes information on all hospitalizations of Manitoba residents, including birth hospitalization information and date of initiation of prenatal care and number of visits abstracted from the prenatal care record.
- Medical Claims/Medical Services file includes information on claims for physician visits, including the service provided, the date of service and a diagnosis code on all ambulatory care contacts for residents of Manitoba, as well as information about physicians’ specialties.
- Drug Program Information Network file includes information on all prescription medications dispensed in the community to Manitoba residents, including prenatal use of prescription medications.
- Manitoba Health Insurance Registry includes information on all Manitobans registered for health care in the province (including demographics such as age of mother and place of residence) and can be used to derive marital status, number of children, and residential postal code, and to determine when residents have moved into or out of the province.
- Canada Census public access file includes area-level sociodemographic information such as average household income, attributed to the population at an aggregate level via the residential six-digit postal code.
- Families First Screen file from Healthy Child Manitoba includes information on 39 social, biological, and demographic risk factors collected by public health nurses within a week of the newborn’s discharge from hospital.
- Employment and Income Assistance data file from Manitoba Families includes information on Manitoba residents who receive support from the Income Assistance Program, a provincial program of last resort for people who need help to meet basic personal and family needs.

A detailed description of the databases can be found online [33].

### Variables

#### Outcome variable: Utilization of prenatal care

The Society of Obstetricians and Gynecologists of Canada (SOGC) recommends that women receive prenatal care visits every 4 to 6 weeks in early pregnancy, every 2 to 3 weeks after 30 weeks’ gestation, and every 1 to 2 weeks after 36 weeks’ gestation [34], while the American Academy of Pediatrics (AAP) and American College of Obstetricians and Gynecologists (ACOG) recommend that women with an uncomplicated first pregnancy be examined every 4 weeks for the first 28 weeks of pregnancy, every 2 to 3 weeks until 36 weeks gestation, and weekly thereafter, while parous women may be seen less frequently [35]. Several indices have been developed to measure the adequacy of prenatal care use, taking into account the month prenatal care began, the number of prenatal visits, and the gestational age at delivery [36, 37]. We selected the Revised Graduated Index of Prenatal Care Utilization (R-GINDEX) [36] for use in this study as it improves on earlier indices and performed well in one of our previous studies [38]. The R-GINDEX is based on the ACOG recommendation for prenatal care visits, and assigns women to one of six categories of care: “no care,” “inadequate,” “intermediate,” “adequate,” “intensive,” and “missing.” For example, at 40 weeks gestation, a woman who began prenatal care in the first 3 months and received between 13 to 16 visits would be categorized as having adequate care, whereas a woman who began care between 1 to 6 months of pregnancy and had less than 8 visits would be categorized as having inadequate care. The intensive care category includes women who have an unexpectedly large number of prenatal care visits, which may indicate potential morbidity or complications.

Information on three birth–related outcomes was used to calculate the R–GINDEX: the gestational age of the infant (obtained from hospital abstracts), the trimester during which prenatal care began, and the total number of prenatal visits during pregnancy. We recorded weeks gestation at the first prenatal care visit and total number of visits from both the hospital abstracts and medical claims files, and used the lower number of weeks gestation and the higher number of visits to reduce the possibility of misclassification of R-GINDEX categories.

### Independent variables

We selected several independent variables that might be associated with utilization of prenatal care based on a review of the literature and availability of variables in the Repository. Maternal age group, young maternal age (< 20 years) at first birth, and parity were obtained from the Hospital Abstracts, while information on maternal education less than grade 12 and a composite variable of smoking, alcohol and/or illicit drug use during pregnancy were obtained from the Families First Screen. Table 1 provides a description of the additional independent variables and how they were defined and calculated. We included selected maternal pre-existing medical conditions and complications of pregnancy because a previous study found that women with medical risks during pregnancy made more prenatal visits [39].

**Table 1:** Description of additional independent variables

Variable	Description
Income Assistance	A woman was considered to have received income assistance if she was coded as having received income assistance anytime during the period of seven months prior to the month of the baby’s delivery to one month after the baby’s delivery (excludes: women living in First Nations communities, stillbirths, out of province births)
Marital Status	
Single Parent	A woman was considered a single (or lone) parent if she was identified as the sole primary care giver for the child on the Families First Screen.
Married or Partnered	A woman giving birth was considered married/partnered if either a marriage was reported to Manitoba Health OR if according to the Families First Screen, she was not a single parent.
Unknown marital status	A woman giving birth was considered to have an unknown marital status if the single parent question on the Families First Screen was left blank or no Families First Screen was done and there was no marriage reported to Manitoba Health.
Income quintile	Income quintiles were developed by assigning average household income from the 2006 Statistics Canada Census to dissemination areas and then ranking these from highest to lowest. Dissemination areas were then grouped into five groups or quintiles (1 being poorest and 5 being wealthiest). Each quintile contained approximately 20% of the population.
Diabetes	A woman was considered to have diabetes if in the three years prior to giving birth she had: 1) one or more hospitalizations with diagnosis code 250 (ICD–9–CM) or E10–E14 (ICD–10–CA) in any diagnosis field over three years of data OR 2) two or more physician claims with diagnosis code 250 over three years of data OR 3) one or more prescriptions for diabetic drugs – Insulins and Analogues (A10A); Blood Glucose Lowering Drugs excluding Insulin (A10BA02, A10BB01, A10BB02, A10BB03, A10BB09, A10BB12, A10BB31, A10BD03, A10BF01, A10BG02, A10BG03, A10BX02, A10BX03) over three years of data OR 4) one or more hospitalizations with gestational diabetes code in the gestation period (ICD–9–CM: 648.8, ICD–10–CA: O24)
Hypertension	A woman was considered to have hypertension if in the one year prior to giving birth she had: 1) at least one physician visit or one hospitalization (ICD–9–CM codes 401–405 or ICD–10–CA codes I10–I13, I15) OR 2) two or more prescriptions for hypertension drugs – Antihypertensives (C02AB01, C02AB02, C02AC01, C02CA04, C02CA05, C02DB02, C02DC01, C02KX01, C02LA01, C02LB01, G04CA03); Diuretics (C03AA03, C03BA04, C03BA11, C03CA01, C03CA02, C03CC01, C03DA01, C03DB01, C03DB02, C03EA01); Beta Blocking Agents (C07AA02, C07AA03, C07AA05, C07AA06, C07AA12, C07AB02, C07AB03, C07AB04, C07AB07, C07AG01, C07BA05, C07BA06, C07CA03, C07CB03); Calcium Channel Blockers (C08CA01, C08CA02, C08CA04, C08CA05, C08CA06, C08DA01, C08DB01); Agents Acting on the Renin–Angiotensin System (C09AA01, C09AA02, C09AA03, C09AA04, C09AA05, C09AA06, C09AA07, C09AA08, C09AA09, C09AA10, C09BA02, C09BA03, C09BA04, C09BA06, C09BA08, C09CA01, C09CA02, C09CA03, C09CA04, C09CA06, C09CA07, C09DA01, C09DA02, C09DA03, C09DA04, C09DA06, C09DA07) OR 3) At least one physician visit or one hospitalization in the gestation period (ICD–9–CM code 642 or ICD–10–CA codes O10–O16)
Antepartum hemorrhage	A woman was considered to have had an antepartum hemorrhage by the presence of: 1) One or more hospitalizations (ICD–9–CM 641, 641.0, 641.1, 641.2, 641.3, 641.8, 641.9; ICD 10– CA O44,O45, O46) in the gestation period indicating antepartum hemorrhage OR 2) One or more physician visits (ICD–9–CM 641, 641.0, 641.1, 641.2, 641.3, 641.8, 641.9) in the gestation period indicating antepartum hemorrhage.
Maternal Psychological distress	A woman was considered to have psychological distress if, in the two years prior to giving birth (or hospital discharge in case of a stillbirth), she had: 1) one or more hospitalizations with a diagnosis for depressive disorder, affective psychoses, neurotic depression, or adjustment reaction (ICD–9–CM codes 296.2–296.8, 300.4, 309, 311; ICD–10–CA codes F31, F32, F33, F341, F38.0, F38.1, F41.2, F43.1, F43.2, F43.8, F53.0, F93.0) OR 2) one or more physician visits with a diagnosis for depressive disorder, affective psychoses, or adjustment reaction (ICD–9–CM codes 296, 309, or 311) OR 3) one or more hospitalizations with a diagnosis for anxiety disorders (ICD–9–CM code 300; ICD–10–CA codes F32.0, F34.1, F40, F41, F42, F44, F45.0, F451, F452, F48, F68.0, F99) OR 4) one or more prescriptions for an antidepressant or mood stabilizer (ATC codes N03AB02, N03AB52, N03AF01, N05AN01, N06A) OR 5) one or more physician visits with a diagnosis for anxiety disorders (ICD–9–CM code 300) and one or more prescriptions for an antidepressant or mood stabilizer (ATC codes N03AB02, N03AB52, N03AF01, N05AN01, N06A) OR 6) one or more hospitalizations with a diagnosis for anxiety states, phobic disorders, or obsessive–compulsive disorders (ICD–9–CM codes 300.0, 300.2, 300.3; ICD–10–CA codes F40, F41.0, F41.1, F41.3, F41.8, F41.9, F42) OR 7) three or more physician visits with a diagnosis for anxiety disorders (ICD–9–CM code 300)
Short inter-pregnancy interval	A short inter-pregnancy interval was defined if the time between the last delivery and conception of the most recent pregnancy was less than 12 months, further divided into two categories: (i) of less than 180 days and (ii) 180–365 days. The date of the last delivery was determined from the Manitoba Health Insurance Registry while conception of the most recent pregnancy was determined from the Hospital Abstract Database.
Social isolation	A woman was considered to have social isolation (defined as lack of social support and/or isolation related to culture, language or geography) if this was identified on the Families First Screen.

Note: Manitoba implemented ICD-10-CA/CCC coding classification system in April 2004

### Data analysis

Rates of prenatal care utilization were calculated for each of the five fiscal years, in order to describe and compare the proportion of women in the no care, inadequate, intermediate, adequate, and intensive categories of prenatal care over time. Thereafter, we combined no care with inadequate prenatal care into one variable for the remaining analyses. Geographical comparisons of rates of inadequate prenatal care between regions of the province were conducted, and the Manitoba provincial average was used as the reference point to determine statistically high, similar, or low rates. A linear trend analysis determined if there was a statistically significant trend in rates of inadequate prenatal care over time, using the Cochran-Armitage Trend Test. Statistical significance for all analyses was defined as *p* < 0.05.

Univariable logistic regression analyses were conducted to determine geographic, socio-demographic and pregnancy-related factors associated with inadequate prenatal care (compared to the reference category of intermediate/adequate prenatal care). Unadjusted odds ratios (uORs) and 95% confidence intervals (CI) of the association between each independent variable and the outcome were calculated. Women with intensive prenatal care were excluded from these analyses. We assessed multicollinearity among the independent variables based on variation inflation factors (VIFs) and tolerance levels (TLs), with multicollinearity defined as VIFs > 2.5 and TLs < 0.40 [40]. Variables with significant uORs were entered into multivariable regression models in order to determine adjusted ORs (aORs) and 95% CI. Two multivariable models were generated: one model for all women in the population giving birth from 2004/05 to 2008/09 (after exclusions), and a second model based on a subset of women having the Families First screen, which captures approximately 80% of the population [27]. Because data missing from the Families First screen may not be random, we reported proportions of missing data for these variables and included the missing category in the regression analyses. The *c* statistic, or area under the receiver operating characteristic (ROC) curve, was calculated to measure the ability of the models to correctly classify those with and without inadequate prenatal care [41]. The statistical analyses were conducted using SAS Software Version 9.2 (Copyright © SAS Institute Inc., Cary, NC, U.S.).

Lastly, because some women had more than one delivery during the period of study, we conducted a sensitivity analysis to remove the effect of multiple deliveries (or observations that were not independent). For women with more than one delivery, we randomly selected one delivery per woman and excluded the other deliveries, and then re-ran the multivariable logistic regression analysis.

## Results

### Participants

There were a total of 70,612 deliveries in Manitoba from 2004/05 to 2008/09. We excluded maternal delivery records that could not be linked to a newborn birth record (0.74%), with a recorded gestation out of range, defined as < 18 or > 45 weeks (0.83%), with a recorded birth weight < 400 g and gestation > 22 weeks (0.06%), and with a maternal Personal Health Identification Number (PHIN) not found on Manitoba Health Registry (0.01%) or not covered by Manitoba Health Registry during pregnancy (2.66%). We excluded midwifery cases having a home birth (0.8%) since prenatal care was not well recorded for those cases. We also excluded midwifery cases of mothers delivered in hospital who were missing a prenatal care record (0.06%), because medical claims data could not be used to determine prenatal care visits as midwives are reimbursed via salary. Lastly, we excluded 211 deliveries that were missing data on the variables required to calculate the R-GINDEX category. These exclusions resulted in a final sample size of 68,132 deliveries, of which 927 of the deliveries were multiple births.

### Utilization of prenatal care

From 2004/05 to 2008/09, the rate of no prenatal care ranged from 0.4 to 0.5%, inadequate care from 9.9 to 12.0%, intermediate care from 50.1 to 51.6%, adequate care from 32.2 to 34.1% and intensive care from 3.6 to 4.3% (Table 2).

**Table 2:** Utilization of prenatal care in Manitoba, 2005/06 to 2008/09 (N = 68,132 deliveries)

Category of prenatal care utilization	2004/05 *N* = 12,808 n (%)	2005/06 *N* = 13,216 n (%)	2006/07 *N* = 13,640 n (%)	2007/08 *N* = 14,134 n (%)	2008/09 *N* = 14,334 n (%)	Total *N* = 68,132 n (%)
No prenatal care	68 (0.5)	67 (0.5)	55 (0.4)	62 (0.4)	59 (0.4)	311 (0.5)
Inadequate prenatal care	1273 (9.9)	1363 (10.3)	1513 (11.1)	1646 (11.7)	1726 (12.0)	7521 (11.0)
Intermediate prenatal care	6578 (51.4)	6752 (51.1)	6835 (50.1)	7298 (51.6)	7316 (51.0)	34,779 (51.0)
Adequate prenatal care	4339 (33.9)	4502 (34.1)	4613 (33.8)	4555 (32.2)	4713 (32.9)	22,722 (33.3)
Intensive prenatal care	550 (4.3)	532 (4.0)	624 (4.6)	573 (4.1)	520 (3.6)	2799 (4.1)

Overall, 11.5% of women had either no care or inadequate prenatal care (hereafter referred to as a combined variable of inadequate prenatal care), and there was a significant increase in the rate of inadequate prenatal care from 10.5 to 12.5% over time (Table 3). Three-quarters (74.5%) of women initiated prenatal care in the first trimester, 22.7% in the second trimester, and 2.6% in the 3rd trimester, while overall 0.5% of women did not receive any prenatal care.

**Table 3:** Rate of combined no care and inadequate prenatal care in Manitoba, 2005/06 to 2008/09 (*N* = 68,132 deliveries)

Category of prenatal care utilization	2004/05 *N* = 12,808 n (%)	2005/06 *N* = 13,216 n (%)	2006/07 *N* = 13,640 n (%)	2007/08 *N* = 14,134 n (%)	2008/09 *N* = 14,334 n (%)	Total *N* = 68,132 n (%)
Inadequate and no prenatal care*	1341 (10.5)	1430 (10.8)	1568 (11.5)	1708 (12.1)	1785 (12.5)	7832 (11.5)

*The rate significantly increased over time (*p* < .0001) based on Cochran-Armitage Trend Test

### Regional variation in prevalence of inadequate prenatal care

**Fig. 1:**
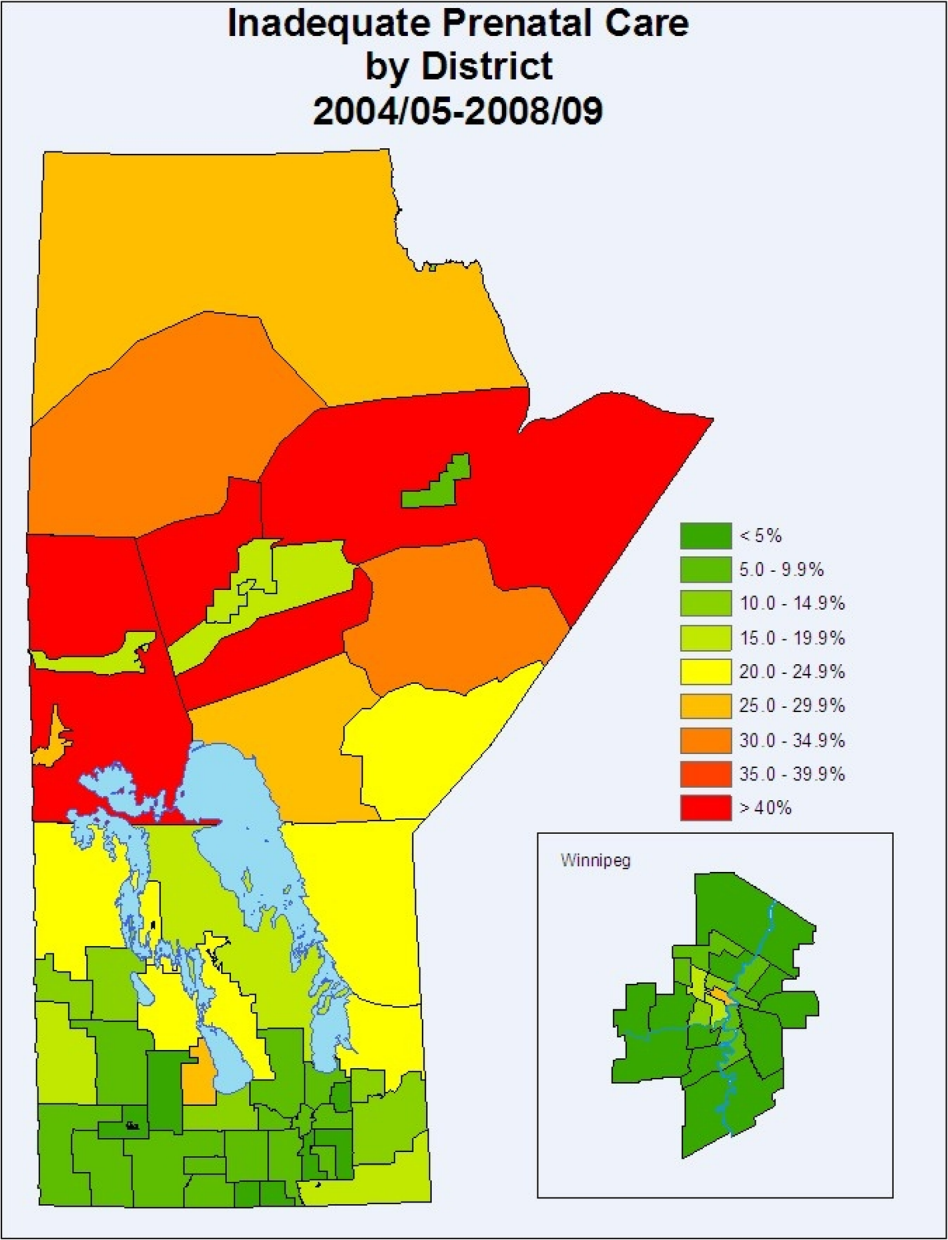
Rates of inadequate prenatal care by geographic district for the province of Manitoba and the capital city of Winnipeg, 2004/05 to 2008/09

**Fig. 2:**
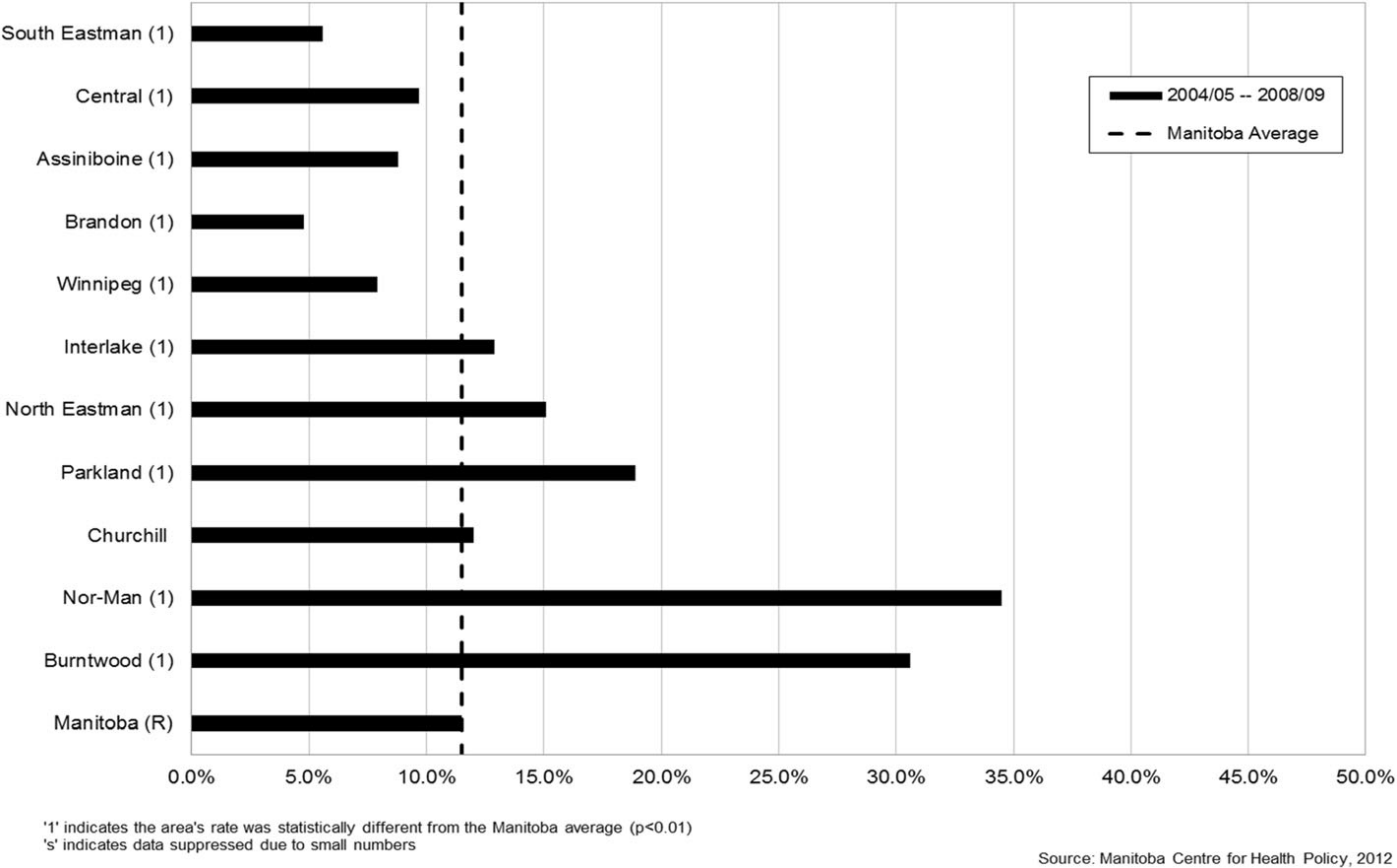
Rates of inadequate prenatal care by regional health authority in the province of Manitoba, 2004/05–2008/09. Regional health authorities are shown in order of socioeconomic status from highest to lowest

**Fig. 3:**
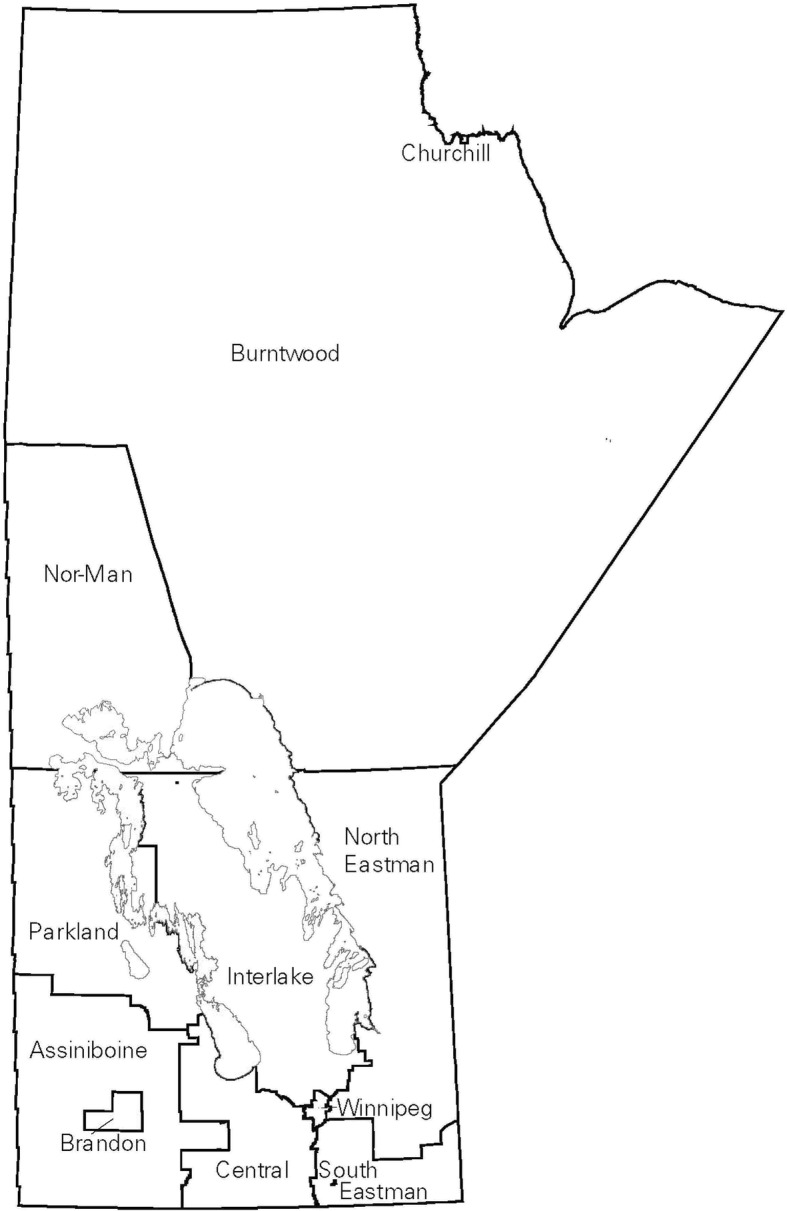
Map of Regional Health Authorities in Manitoba in effect from 2004/05 to 2008/09, corresponding to Fig. 2. Reproduced with permission from the report: Heaman M, Kingston D, Helewa ME, Brownell M, Derksen S, Bogdanovic B, McGowan KL, Bailly A. Perinatal Services and Outcomes in Manitoba. Winnipeg, MB: Manitoba Centre for Health Policy, November 2012

**Fig. 4:**
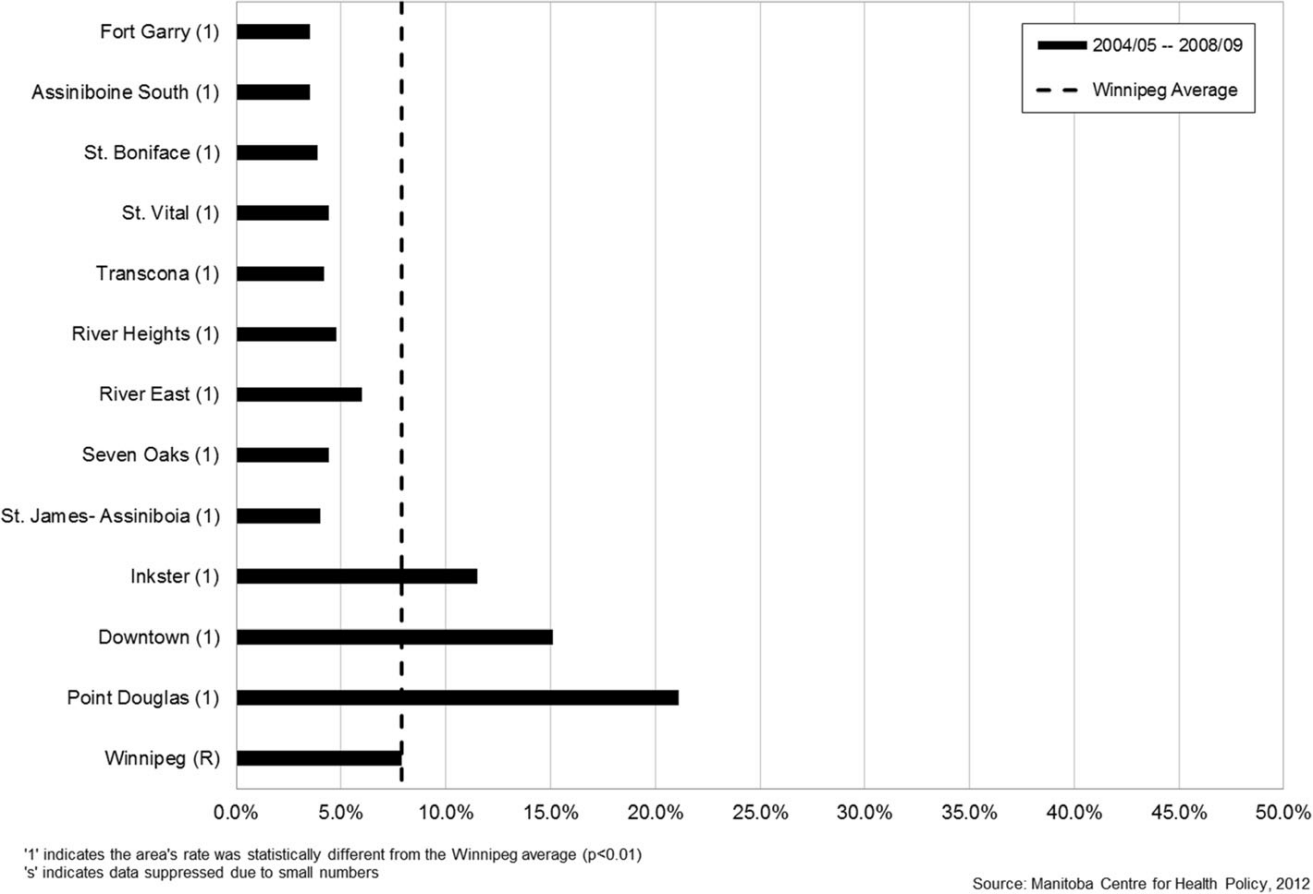
Rates of inadequate prenatal care by community area in the city of Winnipeg, Manitoba, 2004/05–2008/09. Winnipeg community areas are shown in order of socioeconomic status from highest to lowest

**Fig. 5:**
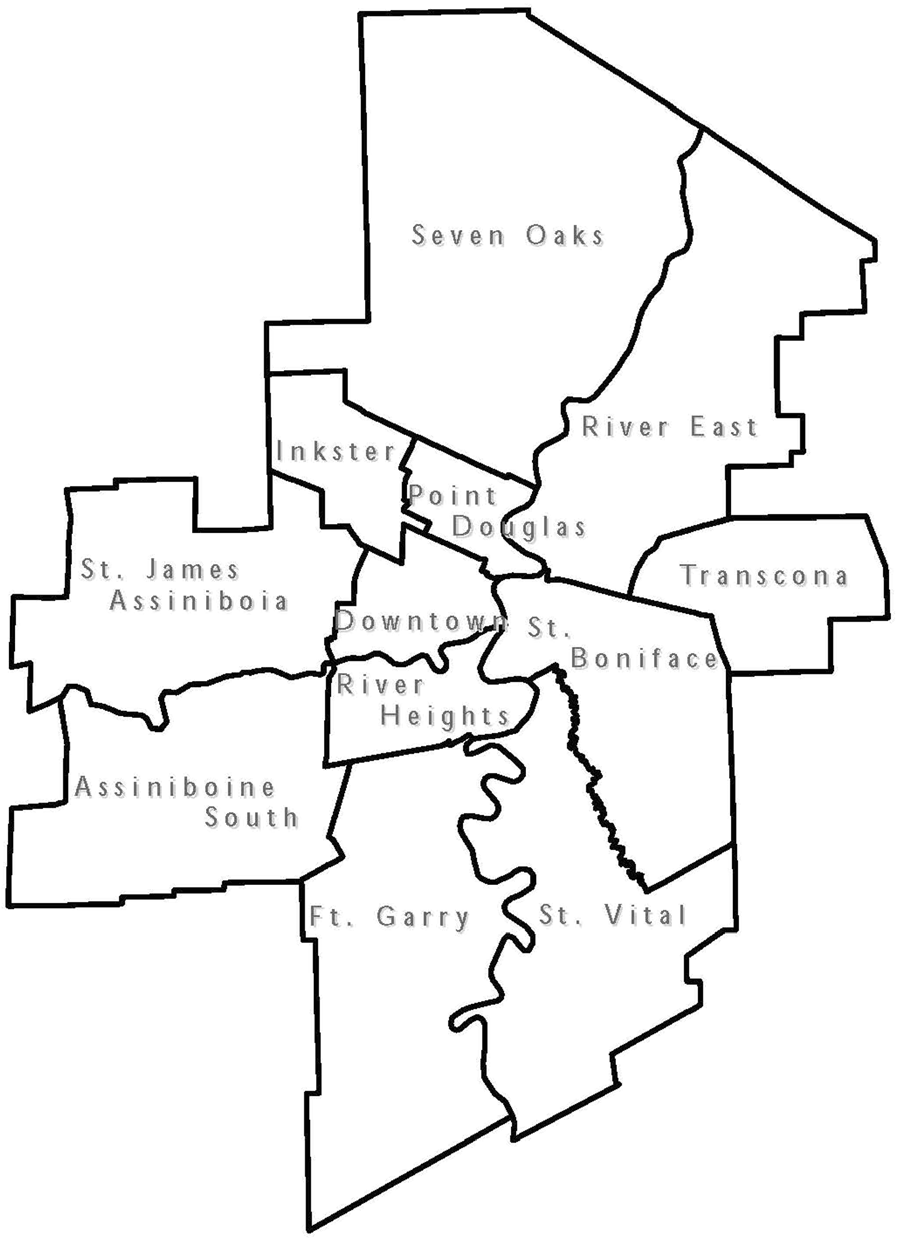
Map of Community Areas in the city of Winnipeg, corresponding to Fig. 4. Reproduced with permission from the report: Heaman M, Kingston D, Helewa ME, Brownell M, Derksen S, Bogdanovic B, McGowan KL, Bailly A. Perinatal Services and Outcomes in Manitoba. Winnipeg, MB: Manitoba Centre for Health Policy, November 2012

There was significant variation in rates of inadequate prenatal care by geographic district across the province and the city of Winnipeg (Fig. 1). The primarily northern regions of Interlake, North Eastman, Parkland, Nor-Man, and Burntwood all had rates of inadequate prenatal care that were significantly higher than the Manitoba average (Figs. 2 & 3). As well, rates of inadequate prenatal care also varied across the Winnipeg community areas, with the inner-city areas of Inkster, Point Douglas, and Downtown having rates that were significantly higher than the Winnipeg average (Figs. 4 & 5).

### Factors associated with Inadequate prenatal care

The proportions of maternal characteristics among deliveries with inadequate prenatal care, adequate/intermediate prenatal care, and intensive prenatal care are presented in Table 4. We excluded deliveries with intensive prenatal care (*n* = 2799) from the regression analyses because a high proportion of women with preexisting conditions or pregnancy complications received intensive care, and these deliveries were therefore judged to be inappropriate to include as part of the reference group. None of the variance inflation factors were > 2.5 (most were < 1.5) and none of the tolerance values were < 0.4 for the variables, indicating that multicollinearity was not a problem in the models.

**Table 4:** Proportions (%) of maternal characteristics among deliveries with inadequate prenatal care, adequate/intermediate prenatal care, and intensive prenatal care for women giving birth in Manitoba from 2004/05 to 2008/09

Characteristic	Entire population of women giving birth (after exclusions) *N* = 68,132			Subset of population of women giving birth who had a Families First screen *N* = 57,603		
	Inadequate/ No prenatal care *N* = 7832	Intermediate/Adequate prenatal care N = 57,501	Intensive prenatal care N = 2799	Inadequate/No prenatal care *N* = 4482	Intermediate/Adequate prenatal care *N* = 50,566	Intensive prenatal care *N* = 2555
Marital status						
Single parent	18.8	77.0	4.2	18.0	77.8	4.3
Married/partnered	5.4	90.1	4.6	4.7	90.7	4.6
Unknown	30.9	66.7	2.4	23.0	73.8	3.3
On Income Assistance						
Yes	26.2	70.3	3.4	24.2	72.1	3.7
No	8.9	86.9	4.2	4.7	90.7	4.6
Income Quintile -neighborhood						
Q1 (lowest)	21.3	75.2	3.6	14.9	80.7	4.4
Q2	13.2	82.7	4.0	8.0	87.5	4.5
Q3	7.0	87.8	5.2	6.0	88.8	5.3
Q4	5.3	90.6	4.1	5.0	91.0	4.0
Q5 (highest)	4.8	91.3	3.9	4.7	91.3	3.9
Maternal Age Group						
12–17 years	27.0	68.9	4.1	22.0	71.4	6.5
18–19 years	24.4	72.7	2.8	18.0	78.5	3.5
20–24 years	16.6	79.8	3.6	11.8	84.0	4.2
25–29 years	9.4	86.7	3.9	6.4	89.4	4.1
30–34 years	6.9	89.0	4.1	4.8	91.0	4.2
35+	7.1	87.0	5.8	5.4	88.6	6.0
Region of Residence						
North	31.4	66.6	2.0	19.3	78.2	2.5
South Rural	10.8	85.3	3.9	6.7	89.2	4.1
Urban (Winnipeg/Brandon)	7.7	87.7	4.7	7.6	87.7	4.7
Maternal Age at First Birth						
< = 19 years	25.4	71.5	3.1	19.4	76.8	3.8
20+ years	6.1	89.4	4.5	4.6	90.8	4.6
Number of births						
Multiple birth	5.2	83.1	11.7	4.1	83.5	12.4
Singleton birth	11.6	84.4	4.0	7.8	87.8	4.3
Parity						
0–3	9.9	85.9	4.2	6.8	88.7	4.5
4+	31.7	65.9	2.4	26.4	70.9	2.7
Inter-Pregnancy Interval						
< 180 days	35.6	62.8	1.6	28.3	70.0	1.8
180–365 days	20.3	77.5	2.3	14.5	83.1	2.5
366+ days	10.9	85.3	3.8	7.7	88.4	3.9
First child	7.5	87.2	5.3	5.1	89.3	5.6
Diabetes						
Yes	8.6	81.7	9.7	4.4	84.5	11.1
No	11.7	84.6	3.7	8.0	88.0	4.1
Hypertension						
Yes	7.1	82.3	10.7	4.3	84.4	11.4
No	11.9	84.6	3.5	8.1	88.1	3.7
Antepartum hemorrhage						
Yes	9.9	82.0	8.0	6.8	84.3	8.9
No	11.6	84.5	3.9	7.8	88.0	4.2
Maternal psychological distress						
Yes	10.6	82.9	6.5	8.2	85.0	6.9
No	11.6	84.6	3.7	7.7	88.3	4.0
Education						
Less than Grade 12	–	–	–	17.2	78.9	3.9
High school or better	–	–	–	3.5	91.8	4.7
Unknown	–	–	–	15.0	81.2	3.8
Social isolation						
Yes	–	–	–	10.9	83.7	5.4
No	–	–	–	5.8	89.6	4.6
Unknown	**–**			16.5	79.9	3.6
Smoking, alcohol and/or illicit drug use during pregnancy						
Yes	–	–	–	12.6	83.0	4.4
No	–	–	–	4.2	91.3	4.6
Unknown	–	–	–	17.3	78.9	3.7

In the first model of all deliveries in the population (*N* = 64,166), shown in Table 5, women were significantly more likely to receive inadequate prenatal care if they lived in the northern (aOR 2.72) or south rural (aOR 1.15) regions of the province compared to the urban areas (the major cities of Winnipeg and Brandon). Women in younger age groups had higher odds of inadequate prenatal care (12–17 years, aOR 1.96; 18–19 years, aOR 1.60; 20–24 years, aOR 1.32) compared to the reference category of 25–29 years, while those 30–34 years had lower odds of inadequate prenatal care (aOR 0.90). Women who were less than or equal to 19 years at their first birth were also at higher odds of inadequate prenatal care (aOR 1.38) compared to women whose first birth was at age 20 or higher. Women who lived in census dissemination areas with an average household income in the 3 lowest income quintiles had higher odds of inadequate prenatal care, compared to those who lived in an area with the highest income quintile. At an individual level, women receiving income assistance had over twice the odds (aOR 2.15) of receiving inadequate prenatal care than women who were not on income assistance. Women were also more likely to have inadequate prenatal care if they were a single parent (aOR 1.85), had a parity of 4 or higher (aOR 2.29), or a short inter-pregnancy interval of either less than 180 days (aOR 3.11) or 180–365 days (aOR 2.26). A variety of medical conditions contributing to an at-risk pregnancy were associated with lower odds of inadequate prenatal care: multiple birth (aOR 0.40), diabetes (aOR 0.47), hypertension (aOR 0.76), antepartum hemorrhage (aOR 0.71), and maternal depression or anxiety (AOR 0.80). The *c* statistic for the first model was 0.83, indicating that the model explained 83% of the area under the ROC curve. Therefore the model had good ability to correctly classify those with and without inadequate prenatal care.

**Table 5:** Factors associated with inadequate prenatal care among women giving birth in Manitoba from 2004/05 to 2008/09, compared to women having intermediate or adequate prenatal care, using multivariable logistic regression (adjusted odds ratios [aOR] and 95% confidence intervals [CI])

Variable	Model 1* (*N* = 64,166)	Model 2** (*N* = 55,048)
	aOR (95% CI)	aOR (95% CI)
Marital status		
Single parent	1.85 (1.69–2.02)	1.47 (1.33–1.63)
Married/partnered	Reference	Reference
Unknown	3.08 (2.89–3.03)	2.24 (1.93–2.59)
On Income Assistance		
Yes	2.15 (2.00–2.30)	1.81 (1.65–1.98)
No	Reference	Reference
Income Quintile -neighborhood		
Q1 (lowest)	1.92 (1.72–2.13)	1.60 (1.14–1.82)
Q2	1.65 (1.47–1.84)	1.34 (1.18–1.52)
Q3	1.27 (1.13–1.44)	1.12 (0.99–1.28)
Q4	1.03 (0.91–1.17)	0.98 (0.86–1.12)
Q5 (highest)	Reference	Reference
Age Group		
12–17 years	1.96 (1.70–2.27)	2.04 (1.68–2.48)
18–19 years	1.60 (1.41–1.80)	1.50 (1.28–1.76)
20–24 years	1.32 (1.22–1.43)	1.31 (1.19–1.44)
25–29 years	Reference	Reference
30–34 years	0.90 (0.83–0.98)	0.89 (0.80–0.98)
35+	0.98 (0.88 1.10)	0.99 (0.87–1.12)
Region of Residence		
North	2.72 (2.52–2.98)	2.53 (2.21–2.98)
South Rural	1.15 (1.41–1.62)	1.30 (1.19–1.41)
Urban (Winnipeg/Brandon)	Reference	Reference
Maternal Age at First Birth		
< 20 years	1.38 (1.28–1.49)	1.32 (1.20–1.45)
20+ years	Reference	Reference
Number of births		
Multiple birth	0.40 (0.29–0.56)	0.52 (0.35–0.75)
Singleton birth	Reference	Reference
Parity		
0–3	Reference	Reference
4+	2.29 (2.09–2.50)	2.59 (2.31–2.91)
Inter-Pregnancy Interval		
< 180 days	3.11 (2.79–3.48)	3.47 (3.01–4.01)
180–365 days	2.26 (2.06–2.50)	2.45 (2.17–2.77)
366+ days	1.48 (1.37–1.61)	1.53 (1.39–1.69)
First child	Reference	Reference
Diabetes		
Yes	0.47 (0.41–0.53)	0.50 (0.41–0.61)
No	Reference	Reference
Hypertension		
Yes	0.76 (0.66–0.85)	0.72 (0.62–0.83)
No	Reference	Reference
Antepartum hemorrhage		
Yes	0.71 (0.63–0.81)	0.71 (0.60–0.83)
No	Reference	Reference
Maternal psychological distress		
Yes	0.80 (0.79–0.85)	0.76 (0.69–0.83)
No	Reference	Reference
Education		
Less than Grade 12	–	1.93 (1.76–2.12)
High school or better	–	Reference
Unknown	–	1.53 (1.33–1.76)
Social isolation		
Yes	–	1.21 (1.03–1.42)
No	–	Reference
Smoking, alcohol and/or illicit drug use during pregnancy		
Yes	–	1.43 (1.31–1.56)
No	–	Reference
Unknown	–	1.03 (0.87–1.23)

*Model 1: Entire population of women giving birth in Manitoba (after exclusions). Value of c statistic for Model 1 = 0.83

**Model 2: Subset of population of women giving birth in Manitoba who had a Families First screen. Value of c statistic for Model 2 = 0.81

In a second model incorporating deliveries which had Families First screening data (*N* = 55,048), the previous factors associated with inadequate prenatal care remained significant, with additional significant factors consisting of maternal education of less than high school (aOR 1.93), social isolation (aOR 1.21), and the composite variable of smoking, alcohol, and/or illicit drug use during pregnancy (aOR 1.43) (Table 5). The c statistic for this model was 0.81.

### Sensitivity analysis

There were 52,144 women and 68,132 deliveries in our original analysis, with 27% of the women having more than one delivery in the five year time frame. After randomly selecting one delivery per woman and re-running the first model (*N* = 48,925), the results remained similar, with aORs of similar magnitude and significance (results available upon request). The only exception was that the aOR for age group 30–34 years became non-significant in the sensitivity analysis (aOR 0.906, 95% CI 0.818–1.004).

## Discussion

The results of this study describe patterns of utilization of prenatal care in the Canadian province of Manitoba, confirm that inequities in use of prenatal care persist, and identify factors associated with inadequate prenatal care that will help inform policy makers and program planners about which populations and regions are most at-risk for inadequate prenatal care. These findings fill an important gap in knowledge related to utilization of prenatal care in Canada, given the lack of surveillance data on prenatal care at a national level in this country.

In terms of utilization, our findings showed that a high proportion of women in Manitoba (11.5%) had inadequate prenatal care, and the rate significantly increased over time from 10.5 to 12.5% during 2004/05 to 2008/09. This rate of 11.5% is higher than that of 6.9% reported in our earlier study [9], which may be a result of using different prenatal care utilization indices – GINDEX [42] versus R-GINDEX [36] - and of improvements in capturing prenatal care utilization in the administrative databases. The higher rates may also reflect changes in provision of health care (e.g., fewer family physicians providing prenatal care) [29] and population trends (e.g., higher proportion of immigrants) [43], although the exact reasons require further exploration. Our population-based rate of 11.5% is much higher than the 4.1% rate of inadequate prenatal care reported by Debessai et al. [17] using data from the Canadian Maternity Experiences Survey [44]. The lower rate reported by Debessai et al. was based on self-report data from a survey of 6421 women in Canada, which may be prone to recall bias, as women may have overestimated their use of prenatal care, and selection bias, as women most at risk of inadequate prenatal care may not have participated in the survey. Findings from the Canadian Maternity Experiences Survey do, however, provide some explanation for the high rates of inadequate prenatal care in Manitoba, as Manitoba had the highest proportion of women who reported not getting prenatal care as early as they wanted (18.6%) compared to the other provinces [44].

Studies from the U.S. and Europe found that a lack of health insurance was an important risk factor for inadequate prenatal care [45, 46]. Somewhat surprisingly, given our universal health care system, the Manitoba rate of inadequate prenatal care of 11.5% was similar to the rate of 11.2% reported in the U.S. using data from 2004 [4]. However, caution needs to be used in comparing these rates because we used the R-GINDEX, whereas the U.S. rate was calculated from birth certificate data using the Adequacy of Prenatal care Utilization Index (APNCU) [47], and rates vary depending on which index is used [47]. Although women in a universal health care system do not have to pay for prenatal care visits, other economic, psychosocial, attitudinal and structural barriers have been shown to negatively influence access to care among women in Manitoba, such as stress and family problems, having an unplanned pregnancy, the costs of transportation and child care, not knowing where to get care or having a long wait for care, and fear of apprehension of the infant by the child welfare agency [18]. Similar barriers have been reported in other studies [48–50], suggesting that health insurance is only one of many factors influencing use of prenatal care. However, only 0.5% of women in Manitoba had no prenatal care, providing evidence that the majority of women (95.5%) accessed at least some prenatal care. Our rate of 0.5% is lower than the rate of 1.0% of women who had no prenatal care in a hospital register based study in Finland, another country which offers free prenatal care [51]. Our Manitoba rate of no prenatal care was also lower than the U.S. rates of 1.9% in 2008 [52] and 1.6% in 2016 [5].

 Our findings showed wide variation in rates of inadequate prenatal care across geographic regions in Manitoba, indicating the persistence of inequities in use of prenatal care similar to the findings of our previous study [9]. The northern regions of the province and inner-city areas in Winnipeg continued to have the highest rates of inadequate prenatal care, and are known to be more socioeconomically deprived. In addition, northern or rural residence was a significant independent factor associated with inadequate prenatal care in the regression models. Possible reasons for this finding might include less access to health care services and prenatal care providers in northern and rural areas of the province, compounded by problems of distance to travel for care. Utilization of prenatal care also followed a clear social gradient, with rates of inadequate prenatal care steadily increasing from a low of 4.8% in the most affluent neighborhoods (income quintile 5) to a high of 21.3% in the poorest neighborhoods (income quintile 1). Living in a neighborhood with the lowest average household income was associated with almost twice the odds (aOR=1.92) of inadequate prenatal care compared to living in a neighborhood with the highest average household income.

Inadequate prenatal care was associated with several individual-level indicators of social disadvantage, such as low income (receiving income assistance), education less than high school, being a single parent, and being assessed as socially isolated. This association between inadequate prenatal care and social disadvantage is similar to findings from other developed countries such as New Zealand [53], England [54, 55] and Belgium [39] and with findings of a systematic review of determinants of prenatal care in high income countries [16]. We also found that young maternal age, high parity, and smoking, alcohol or drug use were factors associated with inadequate prenatal care, congruent with the findings of other studies [11, 16, 39].

To our knowledge, two of our variables have not been studied in previous work and add new knowledge on factors associated with inadequate prenatal care: short inter-pregnancy interval and young maternal age (< 20 years) at first birth. Birth spacing can be measured using inter-pregnancy interval, defined as the time between the last delivery and conception of the current pregnancy [56, 57]. Our results showed that a short inter-pregnancy interval of either less than 180 days, or between 180 and 365 days were both associated with an increased odds of inadequate prenatal care (aOR of 3.11 and 2.26 respectively). Women with closely spaced pregnancies may lack the time or energy to seek prenatal care due to child care responsibilities, or may view prenatal care as unnecessary given the short time since the previous pregnancy. Young maternal age (< 20 years) at first birth was associated with increased odds of inadequate prenatal care (aOR = 1.38). Although young maternal age at first birth is likely at least partly a proxy for lower socioeconomic status, and is associated with number of children in the family, its independent association with inadequate prenatal care in the multivariable regression analyses in this study demonstrates having her first child at a young age may continue to influence a woman’s prenatal care utilization in subsequent pregnancies. In other research, young maternal age at first birth was associated with increased risks of poor health, social and education outcomes among children of prior teen mothers, similar to risks found for children of teen mothers [58].

Our findings also showed that medical conditions such as multiple birth, hypertensive disorders, antepartum hemorrhage, diabetes, and prenatal psychological distress were associated with lower odds of inadequate prenatal care, which suggests that pregnant women with medical risks may seek out more prenatal care, or may have more prenatal care due to increased follow-up and/or referrals to specialists, or more prenatal care may have led to more diagnoses. A higher proportion of women with these conditions received intensive prenatal care compared to those without the condition, as shown in Table 4. Similarly, Beeckman et al. [39] found that women with medical risks during pregnancy made 12% more prenatal visits compared to those without medical risk, while Petrou [59] reported that pregnant women in England and Wales with high risk status at booking had slightly more visits. A study conducted by Krans et al. [60] in Michigan showed that women with high medical risk pregnancies and dual high medical and high psychosocial risk pregnancies were more likely to receive “adequate plus” prenatal care. However, high psychosocial risk pregnancies were more likely to receive inadequate prenatal care.

### Strengths and limitations of the study

This study has several strengths. This study used administrative data to describe utilization of prenatal care and factors associated with inadequate care for the population of women giving birth in Manitoba. Linked administrative databases are a powerful resource for studying important public health issues [30]. However, one important limitation of administrative data is the frequent lack of individual-level socioeconomic information [30]. We were able to overcome that limitation through using highly reliable individual-level information on receipt of income assistance, in addition to ecologic measures such as area-based household income. We were also able to assess social and health behavior factors recorded in the Families First screen.

However, our study also has limitations. This was an observational study, so cause and effect cannot be inferred. In the multivariable regression analyses, multiple individual comparisons could lead to Type 1 error, creating a potential limitation regarding any single factor being studied. In addition, administrative data may be subject to a certain degree of coding errors and incomplete data, which may be random or contain systematic biases. For example, the Families First screening data were available for approximately 80% of the population, and excluded women living in First Nations communities and women having a stillbirth. The completeness of data on number of prenatal visits may be lower for women in some isolated northern communities or other locations where they may be served by salaried physicians, resulting in an over-estimation of rates of inadequate prenatal care.

We selected the R-GINDEX to categorize prenatal care utilization, which is one of several available indices. As previously described, the R-GINDEX is based on the ACOG recommendations for number of visits for low risk pregnant women. Alexander and Kotelchuck note that the effectiveness of this standard has not been assessed through rigorous scientific testing, nor has adequacy of care for women with high risk pregnancies been operationalized [61]. The R-GINDEX is strictly a measure of utilization and only reflects the quantity of prenatal care; it does not measure the content, clinical adequacy, or quality of prenatal care. As well, inaccurate ascertainment of gestational age may affect assignment to a prenatal care utilization category. Our measure of prenatal care also did not take into account use of other maternal health services which may supplement prenatal care, such as participation in the Healthy Baby community support program or prenatal classes.

We were unable to examine maternal characteristics such as unplanned pregnancy, stress and homelessness, which were not captured in the administrative databases. In addition, this study was limited to women having a hospital birth, and excluded the small proportion of women having a home birth with a midwife (0.8%) due to lack of reliable information on number of prenatal visits from the midwifery data. We used firstborn child as the reference category for interpregnancy interval in order to include the full spectrum of birth orders and retain primiparous women in the analysis, based on work by Auger and colleagues [56]. We recognize that some investigators consider the appropriate unexposed category to be women with longer interpregnancy intervals, particularly for studies examining the association between interpregnancy interval and birth outcomes [57]. Lastly, although other studies have found that immigrant women [17, 62] and First Nations women [19, 63] are at higher risk of inadequate prenatal care, the Repository does not include individual-level information on race/ethnicity or immigrant status, so we were unable to study the association of these factors with use of prenatal care. Caution needs to be used in generalizing the results of this study to other Canadian provinces which may have different proportions of First Nations and immigrant women in the population than Manitoba, and different proportions of types of prenatal care providers.

### Implications for practice

Marmot contends that universal health coverage is an important step toward improving access to primary health care, but will not by itself reduce health inequities without also taking action on the social determinants of health [64]. The results of this study confirm that several social determinants of health are associated with inadequate use of prenatal care, such as low income, low education, and rural or northern region of residence. Work to improve social determinants of health needs to be done both within the health sector, and through complementary activities outside health care related to housing, income, education and employment [64]. The Chief Public Health Officer of Canada [1] emphasized the need to address the broader social issues affecting pregnant women, such as low income, homelessness, and substance use, and stated, “Programs that work to break down barriers to prenatal care through community outreach have shown some success through targeting distressed communities and individuals” (p. 52).

Public health interventions to improve prenatal care utilization are important because of the potential to reduce unfavorable births outcomes [12]. Studies in the provinces of Manitoba and Newfoundland have shown that participation in prenatal support programs may improve birth outcomes [24, 25, 65]. Handler and Johnson [66] refer to prenatal care as “a critical anchor of the reproductive/perinatal health continuum for women who do become pregnant, often providing a woman’s first encounter with the health care delivery system” (p. 2221) The factors associated with inadequate prenatal care in this study offer some direction for improving use of prenatal care through strategies such as reduction of teenage pregnancy, optimal birth spacing, cessation of smoking and drug abuse, provision of social support, and providing an income supplement during pregnancy such as the Manitoba Prenatal Benefit [25]. Other authors have recommended paying special attention to socially vulnerable women to reduce variations in use of prenatal care [39, 67] or more systematic attention to the roles of social disadvantage [68], and using a multidisciplinary approach [69]. In Manitoba, we have built on the results of our previous work [9, 18, 70, 71] by implementing health system improvements to reduce inequities in access to and use of prenatal care in inner-city Winnipeg [72, 73].

## Conclusion

Inequities exist in utilization of prenatal care in the province of Manitoba, with wide variations in rates of inadequate prenatal care across geographic regions. Inadequate prenatal care was associated with several individual indicators of social disadvantage, such as low income, education less than high school, and social isolation. Knowledge of these inequities in utilization of prenatal care will help inform policy makers and program planners about which regions and populations are most at-risk for inadequate prenatal care and assist with development of initiatives to reduce inequities in utilization of prenatal care.

## Abbreviations

AAP: American Academy of Pediatrics
ACOG: American College of Obstetricians and Gynecologists
aOR: Adjusted Odd Ratio
APNCU: Adequacy of Prenatal Care Utilization Index
CI: Confidence Interval
GINDEX: Graduated Index of Prenatal Care Utilization
HIPC: Health Information Privacy Committee
MCHP: Manitoba Centre for Health Policy
PHIN: Personal Health Identification Number
R-GINDEX: Revised Graduated Index of Prenatal Care Utilization
ROC: Receiver operating characteristic
SOGC: Society of Obstetricians and Gynecologists of Canada
TL: Tolerance Levels
U.S.: United States
uOR: Unadjusted Odd Ratio
VIF: Variation Inflation Factors
